# The Role of Metabolic Syndrome in Endometrial Cancer: A Review

**DOI:** 10.3389/fonc.2019.00744

**Published:** 2019-08-08

**Authors:** Xiao Yang, Jianliu Wang

**Affiliations:** Department of Obstetrics and Gynecology, Peking University People's Hospital, Beijing, China

**Keywords:** endometrial cancer, metabolic syndrome, metabolic microenvironment, signaling pathway, targeted therapy

## Abstract

Endometrial cancer is one of the most common cancers of the female reproductive system. Although surgery, radiotherapy, chemotherapy, and hormone therapy can significantly improve the survival of patients, the treatment of patients with very early lesions and a strong desire to retain reproductive function or late recurrence is still in the early stages. Metabolic syndrome (MS) is a clustering of at least three of the five following medical conditions: central obesity, high blood pressure, high blood sugar, high serum triglycerides, and low serum high-density lipoprotein (HDL). Obesity, diabetes and hypertension often coexist in patients with endometrial cancer, which increases the risk of endometrial cancer, also known as the “triple syndrome of endometrial cancer.” In recent years, epidemiological and clinical studies have found that MS associated with metabolic diseases is closely related to the incidence of endometrial cancer. However, the key molecular mechanisms underlying the induction of endometrial cancer by MS have not been elucidated to date. Characterizing the tumor metabolism microenvironment will be advantageous for achieving a comprehensive view of the molecular mechanism of metabolic syndrome associated with endometrial cancer and for providing a new target for the treatment of endometrial cancer. This review focuses on recent advances in determining the role of metabolic syndrome-related factors and mechanisms in the pathogenesis of endometrial cancer. We suggest that interfering with the tumor metabolic microenvironment-related molecular signals may inhibit the occurrence of endometrial cancer.

## Introduction

Endometrial cancer is one of the most common gynecological malignancies. The latest cancer statistics from the American Cancer Society showed that in 2018, the number of new cases of endometrial cancer in the United States was 63,230, and the number of deaths was 11,350. The incidence of malignant endometrial tumors in women ranked fourth, and the incidence of death from endometrial cancer ranked sixth (1). With the increasing incidence of metabolic diseases (obesity, diabetes and hypertension), the incidence of endometrial cancer is increasing, and affecting younger populations worldwide. It is estimated that the incidence of endometrial cancer will increase to 42.13 per 100,000 people in the United States by 2030 (2). In recent years, early diagnosis, surgery, radiotherapy and chemotherapy can significantly improve the therapeutic effect of patients, but the treatment of early lesions and the need to retain fertility, late and recurrent patients is still limited. A clinical analysis of 276 patients with endometrial cancer showed that the 5-year disease-free survival rate and the 5-year overall survival rate were 82.3 and 81%, respectively, and the recurrence rate and the cancer-related mortality rate were 14.5 and 15.9%, respectively (3).

Regarding the pathogenesis of endometrial cancer, the traditional view is that long-term non-progesterone estrogen overstimulation of the endometrium is the main cause of endometrial hyperplasia and endometrial cancer. Estrogen can bind with nuclear estrogen receptor (ER) and play a “genotype”-regulatory effect by regulating the transcription of specific target genes. Additionally, our previous studies have found that estrogen can also induce Ca^2+^ influx by binding to the G protein-coupled estrogen receptor (GPER) on the cell membrane surface, activating the calcium channel Cav1.3, and activating the downstream signal transduction pathway (MAPK/Erk) rapidly, thereby promoting the proliferation of endometrial cancer. This process does not involve gene transcription and protein synthesis; therefore, it is called the “non-gene-transcription effect” (4). At present, long-term progesterone is commonly used in the clinical treatment of endometrial cancer. However, the overall effective rate of progesterone therapy for primary endometrial cancer is only 50–70%, and the recurrence rate is as high as 40% (5, 6). The objective response rate of progesterone therapy for advanced and recurrent endometrial cancer is only 15–20% (7). Interestingly, recent studies have shown that serum estrogen levels in patients with endometrial hyperplasia and endometrial cancer are not elevated compared with those in the normal control group (8). Moreover, epidemiological studies have shown that long-term estrogen exposure in post-menopausal women does not increase the risk of endometrial cancer (9). Traditional views do not explain why endometrial cancer still occurs in post-menopausal women with low estrogen levels. These studies suggest that local estrogen sensitivity, rather than increased circulating estrogen, may drive the occurrence and development of endometrial cancer. At the same time, other factors besides estrogen may also induce the occurrence and development of endometrial cancer.

Endometrial cancer is often associated with obesity, diabetes, and hypertension. These conditions are commonly known as the metabolic triad of endometrial cancer. Epidemiological studies showed that the risk of endometrial cancer in diabetic patients was 2.12 times higher than that in normal patients, while the risk of endometrial cancer in those who were overweight (BMI ≥ 25 kg/m^2^) was 2.45 times higher than that in the control group. The risk of endometrial cancer in obese patients with hypertension was 3.5 times higher than that in the control group. Additionally, endometrial cancer is one of the cancers most closely related to metabolic diseases (10). Several studies have shown that metabolic syndrome caused by obesity, diabetes and hypertension is closely related to the incidence and adverse prognosis of endometrial cancer. A meta-analysis of six studies reported that metabolic syndrome is closely associated with increased risk of endometrial cancer in women (relative risk: 1.89, 95% CI 1.34–2.67) (11). A new research reported that there was a very high prevalence of metabolic syndrome in women newly diagnosed with endometrial cancer (12). A prospective case control study reported that women newly diagnosed with endometrial cancer have a higher prevalence of incident hyperglycemia, total: HDL cholesterol ratio, and three or more cardiovascular risk factors than women without endometrial cancer (13). All these studies suggest that metabolic syndrome is closely related to the incidence of endometrial cancer. However, the exact mechanism of metabolic syndrome affecting the occurrence and development of endometrial cancer has not been determined to date, which may be related to the elevation of such metabolites as blood sugar, insulin, insulin-like growth factor and triglyceride in serum (14, 15). The dynamic interaction between cells and the cell microenvironment plays an important role in regulating the growth of normal tissues and cancer cells. The tumor cell microenvironment includes tumor cells and other cells, such as fibroblasts, lymphocytes, macrophages, adipocytes, and other secreted factors, to form a unique tumor microenvironment system. An abnormal imbalance of the cell microenvironment often leads to tumorigenesis (16). It is suggested that molecules related to metabolic syndrome can accelerate the progression of endometrial cancer not only by acting directly on tumor cells but also by further remodeling the immune microenvironment of tumors.

In this review, we focus on the metabolic microenvironment of endometrial cancer and summarize the key molecular signaling pathways of obesity, diabetes and hypertension-related metabolic syndrome affecting the occurrence, development and prognosis of endometrial cancer, aiming to explore new methods of early prevention, and targeted treatment of endometrial cancer.

## Molecular And Metabolic Mechanisms Underlying the Obesity-Endometrial Cancer Link

**Figure 1:**
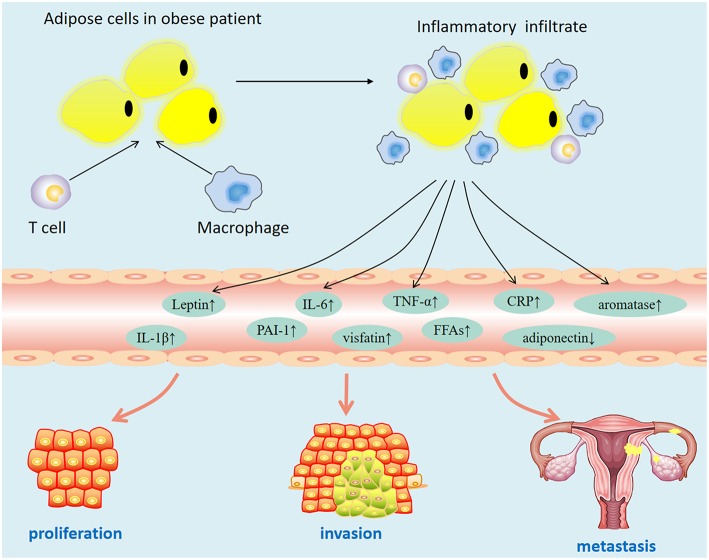
Dysfunctional adipose tissue in obesity.

Epidemiological data showed that obesity is closely related to the increase in the incidence of various cancers. Some scholars have confirmed the causal relationship between obesity and breast cancer by constructing a genetically engineered mouse model and have shown that obesity is closely related to the increase in the survival rate of residual cancer cells (17). A meta-analysis of 26 studies in the United States showed that every five units increase in body mass index (BMI) increased the risk of endometrial cancer by 50% [relative risk [RR], 1.50; 95% CI, 1.42–1.59] (18). A study reported that a history of bariatric surgery and maintained normal weight after surgery is associated with a 71 and 81% reduced risk for uterine malignant tumors (19). These findings suggest that obesity may be a modifiable risk factor related to development of endometrial cancer. However, the mechanism by which obesity increases the risk of endometrial cancer has not been elucidated. At present, the possible mechanisms are as follows: obese patients are often accompanied by insulin resistance (hyperinsulinemia), abnormal fat metabolism (leptin, adiponectin disorders), hyperglycemia, hyperlipidemia, and chronic inflammation. These factors may promote the occurrence and development of tumors (Figure 1). To explore the key mechanism of obesity-induced endometrial cancer occurrence and development, it is helpful to provide a new intervention target for the treatment of patients with metabolic syndrome.

### Adipocyte-Derived Estrogen Signaling

Endometrium is a highly dynamic tissue controlled by ovarian steroids, estrogen and progesterone. Long-term estrogen stimulation without progesterone antagonism is a key factor in the occurrence of endometrial cancer. The decline of ovarian function in post-menopausal women is accompanied by a decrease in hormone levels. However, post-menopausal women are still prone to endometrial cancer. It has been reported that adipose-derived aromatase converts circulating androstenedione into estradiol, leading to elevated serum estradiol levels, which binds to estrogen receptors α and β (ERα and ERβ), eventually leading to recruitment of transcription factors, and gene transcription may be activated or repressed (20). Thus, in post-menopausal women, adipose tissue is the main source of estrogen biosynthesis. In addition, obesity can lead to hyperinsulinemia, which can reduce the synthesis of sex hormone binding protein (SHBG) by increasing the bioavailability of insulin-like growth factor-1 (IGF-1), thereby leading to an increase in estrogen levels. Therefore, obesity increases the risk of endometrial cancer, possibly by indirectly affecting estrogen levels. A meta-analysis showed that hormone replacement therapy (HRT) could modified the BMI-endometrial cancer risk association, however, menopausal status and histologic subtype did not significantly impact upon these associations (21). These findings support the hypothesis that hyperestrogenia is an important mechanism underlying the BMI-endometrial cancer association. Additional studies are needed to explore the exact mechanism mediating the link between body adiposity and endometrial cancer.

### Adipocyte-Derived Insulin Resistance

In obese patients, excessive accumulation of adipose tissue leads to elevated levels of circulating free fatty acids and increased expression of serum adipokines, such as leptin, visfatin, and cytokines, which ultimately leads to insulin resistance. Hyperinsulinemia with a decrease in the serum level of IGF-1 binding protein and an increase in IGF-1 is most often caused by insulin resistance. Obesity-induced chronic low-grade inflammation is an important factor leading to insulin resistance. Obesity-related inflammation is characterized by increased macrophage infiltration and increased expression of inflammatory cytokines in adipose tissue (22). Recently, studies have reported that endoplasmic reticulum chaperone 78 (GRP78) plays an important role in obesity-induced insulin resistance by regulating macrophages (23). The level of the proinflammatory cytokine TGF-α in adipose tissue of obese mice was significantly increased, which was closely related to insulin resistance (24). There are a large number of inflammatory mediators, such as C-reactive protein (CRP), interleukin-6 (IL-6), and plasminogen activator inhibitor-1 (PAI-1), that are elevated in the plasma of obese patients or animals and are closely related to insulin resistance (25). The obesity-induced inflammatory response inhibits insulin signaling in adipocytes and hepatocytes through a variety of signaling pathways, including inhibition of the expression of insulin receptor substrate 1 (IRS-1) and insulin receptor (IR) in insulin signaling pathways and inhibition of PPAR gamma function, and ultimately leads to insulin resistance (26, 27). Increased insulin and IGF-1 can stimulate the proliferation of endometrial cancer cells by binding to IR and IGF-1 receptors (IGF-1R) and activating downstream signaling pathways (28).

### Synergistic Interaction of Estradiol and Insulin Signaling

There are both genotypic transcriptional and non-transcriptional effects in estrogen signal transduction: estrogen binds to ERα in the nucleus to exert genotypic effects, and the estrogen receptor GPER is located on the cell membrane to exert non-genotypic transcriptional effects. Studies have shown that estrogen combined with insulin can significantly promote the proliferation of endometrial cancer cells compared with estrogen or insulin alone (29). The combination of estrogen and a high-fat diet (mimic insulin resistance) could significantly stimulate the increase of endometrial glands in C57BL/6 mice. The nuclear factor kappa-light-chain-enhancer of activated B cells (NF-κB), peroxisome proliferator activated receptor (PPAR) and LXR/RXR signaling pathways may be involved in this process (30). Insulin upregulates TET1 and then upregulates GPER expression, which enhances the sensitivity of endometrial cancer cells to estrogen (31). Studies have found that estrogen and IGF-1 can synergistically promote the development of tumors in mice by activating MAPK and AKT signaling pathways (32). Other studies have found that estrogen may bind to IGF-1R and exert non-genetic transcriptional effects through the Ras/MAPK signaling pathway (33). How insulin and IGF-1 interact with estrogen in signaling pathways to promote the development of endometrial cancer warrants further investigation.

### Adipose-Derived mTOR Signaling

Compared with non-obese patients, the activity of VEGF-mTOR in obese endometrial cancer patients increased significantly, suggesting that adipocyte-derived VEGF-mTOR signaling is a potential target for the treatment of obese women with endometrial cancer (34). Adipose tissue mesenchymal stem cell-derived medium can activate Akt/mTOR and promote the proliferation and invasion of cancer cells (35). A new bidirectional inhibitor of PI3K and mTOR is more effective than a simple inhibitor of mTOR (rapamycin) in inhibiting endometrial cancer (36). It is suggested that mTOR signaling may be a key pathway connecting obesity and endometrial cancer, which needs to be verified in a clinical trial.

### Adipose-Derived Stem Cells

Adipose-derived stem cells play an important role in the tumor microenvironment. Obesity can promote the transformation of adipose-derived stem cells (ASCs) into cancer-related fibroblasts (CAFs), thereby promoting the proliferation and invasive phenotype of cancer cells (37). In addition, adipose-derived stem cells can promote ER + breast cancer cell metastasis independently of estrogen signaling (38). Leptin secreted by adipose-derived stem cells promotes the growth and metastasis of ER + breast cancer by increasing the expression of ER receptor and aromatase (39). It has been reported that ASCs can fuse with endometrial cancer cells, and the fused endometrial cancer cells present a fibroblast-like appearance of mesenchymal phenotype accompanied by downregulation of E-cadherin expression and upregulation of Vimentin expression (40). Further study of the role of ASCs in the occurrence of endometrial cancer will help to elucidate how obesity increases the risk of endometrial cancer.

### Adipose-Derived Adipokines

#### Adiponectin

Adiponectin is a cytokine secreted mainly by adipocytes. Epidemiological studies have shown that adiponectin levels in the blood circulation of patients with endometrial cancer are decreased (41). Serum low adiponectin levels are closely related to insulin resistance, hyperinsulinemia, obesity, and hypertension (42). A meta-analysis showed that serum adiponectin levels were negatively correlated with the risk of endometrial cancer, especially in post-menopausal women who did not receive HRT (43). In a meta-analysis of 26 studies, for each 1 μg/ml increase of adiponectin, there was a 3% reduction of summary relative risk (SRR) in endometrial cancer risk, and a 14% reduction for each increase of 5 μg/ml (44). Abnormal expression of serum adiponectin is also closely related to the occurrence and development of prostate cancer, breast cancer and colon cancer (45–47). However, the antitumor effect of adiponectin on endometrial cancer mainly depends on the alteration of systemic metabolic state or the direct interaction with tumor cells warrants further study.

Adiponectin exerts its biological effects mainly by binding to adiponectin receptors. Three types of adiponectin receptors have been identified: adiponectin receptor 1 (AdipoR1), adiponectin receptor 2 (AdipoR2), and T-cadherin. AdipoR1 is mainly expressed in skeletal muscle and epithelial cells, and AdipoR2 is most abundant in the liver (48). AMPK is an important signaling pathway downstream of adiponectin. The adiponectin/AdipoR1 signaling axis can promote the phosphorylation of the AMPK Thr172 site by inducing phosphorylation of the tumor suppressor gene LKB1. AMPK phosphorylation also plays an important role in energy metabolism by activating the TSC2 tumor suppressor (49, 50). Studies have shown that there is no significant difference in the expression of AdipoR1 and AdipoR2 in normal endometrial tissues. In endometrial cancer tissues, the expression of AdipoR1 is higher than that of AdipoR2. Adiponectin/AdipoRs can inhibit the proliferation, adhesion and invasiveness of endometrial cancer cells by activating the downstream LKB1-AMPK/S6 signal axis (51). Adiponectin can not only inhibit the proliferation and migration of endometrial cancer cells through the AMPK/mTOR/S6K1 signaling pathway but can also enhance the sensitivity of endometrial cancer cells to insulin through the AMPK/S6K1/IRS1 signaling pathway (52). Adiponectin increases insulin sensitivity mainly by activating p38MAPK activity. It has been reported that the highly conserved 13-residue segment (ADP-1) of adiponectin can promote the translocation of glucose transporter 4 (GLUT4) to the cell membrane, reduce the blood sugar level of db/db mice and promote the secretion of insulin by pancreatic beta cells, thereby improving the metabolism of glucose and fatty acids (53). In addition, the anti-proliferative effect of adiponectin is related to a variety of cell cycle regulators, cyclin D1, D2, ERK1/2, and Akt.

It is worth noting that adiponectin not only affects the tumor cells themselves but also regulates the tumor immune microenvironment. In contrast, it has been reported that the deletion of adiponectin may promote the transformation of M2 tumor-associated macrophages to M1 type through the p38MAPK signaling pathway, thereby inhibiting the growth of tumors (54). Therefore, on the one hand, adiponectin can reduce the occurrence of endometrial cancer by changing the metabolic state of the whole body; on the other hand, it can directly inhibit the proliferation of endometrial cancer cells. The decrease in serum adiponectin levels in obese patients is closely related to the increased risk of endometrial cancer (44).

#### Visfatin

Visfatin is an insulin-like adipokine identified in recent years. Visfatin is highly expressed in many metabolically related tumors, and its increased expression is closely related to the increased risk of cancer (55). It has been reported that decreased serum adiponectin or increased visfatin levels are independent risk factors for endometrial cancer. The visfatin:adiponectin ratio in the endometrial cancer was significantly higher than the control, which has certain reference value for the diagnosis of endometrial cancer (56). With the increase of BMI, the level of visfatin in obese patients increases significantly. The increase in serum visfatin level is closely related with risk of myometrial invasion (OR: 1.091; 95%CI:1.021–1.166) and lymph node metastasis (OR: 1.018; 95%CI:1.000–1.035) of endometrial cancer. A high level of visfatin suggests poor prognosis in patients with endometrial cancer, which may be a potential therapeutic target for endometrial cancer (57, 58). Studies have shown that visfatin can upregulate the expression of IR and insulin receptor substrate (IRS) 1/2, and it can coactivate the PI3K/Akt and MAPK/ERK1/2 signaling pathways with insulin to promote the proliferation and inhibit apoptosis of endometrial cancer cells (59). Exogenous visfatin can promote the proliferation of breast cancer cells by promoting ERα phosphorylation and activating estrogen response element (ERE)-dependent signaling pathways (60). However, it has not been reported whether visfatin can also enhance estrogen-dependent ER signaling and accelerate the development of endometrial cancer. In addition, visfatin has been reported to be abnormally expressed in a variety of tumors, which can increase the risk of multiple tumors and become a potential molecular marker for the early detection of tumors (55). Therefore, the combination of visfatin and adiponectin may be a marker for the early clinical diagnosis of endometrial cancer and may provide new targets for clinical intervention.

#### Leptin

Leptin is an important adipokine encoded by the obesity gene. Leptin plays an important role in regulating food intake, energy consumption and promoting cell growth by combining with leptin receptor (ObR). Recent studies have found that abnormal expression of leptin and leptin receptor signaling related to obesity plays an important role in the development of breast, colon and endometrial cancer (61, 62). A meta-analysis showed that high levels of leptin can significantly increase the risk of endometrial cancer (risk ratio, RR = 2.55) and that high levels of leptin are an independent risk factor for endometrial cancer (63). The expression of leptin and ObR is positively correlated with the invasiveness of tumors and BMI of patients but is negatively correlated with histological grade. The elevated expression of leptin and ObR is closely related to lymph node metastasis and poor survival prognosis, as well as the positive expression of ERs (64). However, whether the leptin signaling pathway can influence the development of endometrial cancer by affecting the classical estrogen signaling pathway remains to be further confirmed. Studies have shown that the expression of ObR in poorly differentiated endometrial cancer tissues is significantly higher than that in well-differentiated endometrial cancer tissues, and leptin can inhibit the apoptosis of endometrial cancer cells by activating the NIK/IKK signaling pathway. Elevated leptin levels can influence epithelial polarity and promote malignant transformation through overactivation of the PI3K/Akt signaling pathway (65). Leptin also promotes the proliferation and invasion of endometrial cancer cells by activating STAT3 and ERK1/2, JNK signaling pathways. Correspondingly, this proliferation is inhibited when the JAK/STAT3 pathway is blocked (66). Other studies have reported that the leptin-2548 G/A SNP may be involved in the occurrence and development of endometrial cancer (67). It has been reported that leptin can upregulate the expression of STAT3-CPT1B and plays an important role in maintaining the stem and drug resistance of breast cancer cells (68). However, the role of leptin in the maintenance stem cell of endometrial cancer has not been determined to date and requires further study. Elevated leptin levels suggest the presence of endometrial cancer, and serum leptin levels may be an effective tool for assessing the clinical staging of endometrial cancer (69). Although elevated leptin level is a high-risk factor for endometrial cancer, whether it is the most critical molecule associated with obesity and endometrial cancer warrants further investigation.

### Adipose-Derived Inflammatory Cytokines

Inflammation is the core stage of tumorigenesis and development. It has been reported that 18% of cancer cases worldwide are related to chronic infection, which implies a potential relationship between cancer and inflammation. In a lean state, a balance between adipocytes and immune cells can maintain normal metabolism throughout the body. However, in obese people, this balance translates into a markedly inflammatory adipose tissue microenvironment. Obesity-related adipose inflammation can increase the secretion of pro-inflammatory factors, cause systemic metabolic disorders, and change the microenvironment of tumors, thereby significantly increasing the risk of cancer in obese people. Several studies have confirmed that obesity-related inflammatory cytokines are involved in tumorigenesis.

#### IL-6

IL-6 is an inflammatory cytokine that plays an important role in many physiological and pathological processes. IL-6 is closely associated with a three times increased risk of mortality in overweight/obese patients (70). Studies have confirmed that IL-6 is closely related to the occurrence of a variety of tumors, including endometrial cancer. Adipose-derived IL-6 can promote the proliferation, invasion and angiogenesis of endometrial cancer cells by activating the JAK/STAT3 signaling pathway (71). The elevated plasma level of IL-6 is closely related to the poor prognosis of tumors (72). In addition, estrogen can promote the expression of IL-6 in endometrial cancer cells by binding to GPER on the cell surface (73). It has been reported that estrogen (E2) can promote the expression of IL-6 by binding with nuclear receptor ERα, and IL-6 can promote the synthesis of aromatase by binding with IL-6R of basal cells, thereby accelerating the synthesis of estrogen and forming a positive feedback loop (74). A new study confirms that blocking IL-6-driven inflammatory signaling can inhibit the spread of cancer cells to the liver (75). IL-6 is likely to play a prominent role in the development of endometrial cancer, and it appears to be one of the major mechanisms involved in the obesity-cancer link.

#### TNF-α

TNF-α is an inflammatory cytokine secreted by macrophages and adipocytes. It is an important regulator of adipose tissue metabolism and plays an important role in immune regulation, inflammatory response and anti-tumor response. However, recent studies have found that TNF-α is also an endogenous tumor-promoting factor that can promote the proliferation, invasion and metastasis of cancer cells. The level of TNF-α in circulating blood of obese patients is increased, and the increased level of TNF-α is closely related to the poor prognosis of endometrial cancer patients (76). In addition, studies have reported that 11 cancer markers in overweight patients are significantly higher than those in normal weight patients, including ANG-2, sFASL, HB-EGF, IL-8, PLGF, TGF-α, TNF-α, uPA, VEGF-A, VEGF-C, and VEGF-D (77). Compared with lean mice, C57BL/6 mice induced by a high-fat diet had higher levels of serum free fatty acids and TNF-α and higher accumulation of macrophages in adipose tissue (78). A case-control study showed that elevated levels of TNF-α and its soluble receptors (sTNFR1 and sTNFR2) were associated with an increased risk of endometrial cancer [TNF-α-odds ratio [OR]: 1.73; sTNFR1-[OR]:1.68; sTNFR2-[OR]:1.53] (79). Regarding the mechanism of TNF-α in promoting tumorigenesis and development, it has been reported that chronic inflammation induced by obesity promotes the accumulation of macrophages in adipose tissue. TNF-α released by M1 macrophages can promote metastasis and inhibit apoptosis of ovarian cancer cells by activating the signaling pathway of NF-κB (80). TNF-α is also a key factor driving the expression of the aromatase gene, and IL-10 can regulate the expression of aromatase in adipose tissue by inhibiting the TNF-α signaling pathway (81). Moreover, TNF-α could induce serine phosphorylation of IRS-1 and inhibit its triggering of downstream signals, leading to insulin resistance. Insulin resistance-induced hyperinsulinemia and IGF-1 can further enhance the biological effects of TNF-α by activating the TNF-α signaling pathway (80). TNF-α inhibited apoptosis in cancer cells by activating the NF-κB signaling pathway. Although several pathways between TNF-α and tumors have been identified, the precise mechanism of obesity-related TNF-α involved in the development of endometrial cancer remains to be further investigated.

#### PAI-1

Plasminogen activator inhibitor-1 (PAI-1) is a protease inhibitor produced by vascular endothelial cells, stromal cells and adipocytes in adipose tissue. Recent studies have found that PAI-1 not only plays an important role in influencing insulin signaling but also plays an important biological role in influencing the invasion, invasion and metastasis of obesity-related tumors (82). PAI-1 is highly expressed in endometrial cancer tissues and is closely related to the poor prognosis of endometrial cancer (83). Studies have reported that PAI-1 may mediate the transcriptional regulation of adipose-derived stem cells in endometrial cancer (84). Therefore, PAI-1 represents a potential therapeutic target.

### Effect of Obesity on the Tumor Immune Microenvironment

Obesity can promote adipocytes to secrete pro-inflammatory factors, such as TNF-α, IL-6, and IL-18. These pro-inflammatory cytokines can further enhance the infiltration of inflammatory cells, mainly macrophages and T lymphocytes, thereby promoting abnormal proliferation and transformation of normal cells (85). Studies have shown that 16 weeks of aerobic and endurance training can reduce the expression of inflammatory cytokines (IL-6 and TNF-α) in adipose tissue and induce the transformation of inflammatory M1 macrophages into anti-inflammatory M2 macrophages (86). It has been found that adipose tissue-derived leptin can promote the differentiation of Th17 cells and promote T cell function by regulating cell metabolic reprogramming (87). Recent studies have shown that obesity can increase the infiltration of tumor-related macrophages, upregulate the production of IL-1β, and promote angiogenesis and tumor progression (88). It has been reported that M2 macrophages infiltrated in the microenvironment of endometrial cancer can enhance the sensitivity of endometrial cancer cells to estrogen by releasing cytokine IL17A and upregulating the expression of ERα through TET1-mediated epigenetics (8). These studies indicated that obesity may further promote the occurrence and progression of endometrial cancer by affecting the immune microenvironment of tumors.

## Type 2 Diabetes And Endometrial Cancer

Most epidemiological studies suggested that diabetes is a risk factor for endometrial cancer incidence; for example, a meta-analysis of 16 studies showed that diabetes was statistically significantly associated with an increased risk of endometrial cancer (summary RR 2.10, 95% CI 1.75–2.53), and there was a stronger association with a adjusting for age (RR 2.74, 95% CI 1.87–4.00) (89). Also, diabetes is closely related to increased cancer-specific mortality (HR 2.09, 95% CI 1.31–3.35) and mortality from non-cancer related causes in women with endometrial cancer (90). Therefore, these studies show that diabetes increases both the risk and mortality rates of endometrial cancer.

### Effect of Hyperglycemia on Endometrial Cancer

Hyperglycemia is an important clinical characteristic of type 2 diabetes mellitus. Systemic hyperglycemia provides favorable conditions for energy metabolism of cancer cells. Previous studies have confirmed that elevated serum glucose can directly regulate cancer-related signaling pathways, especially to meet the needs of rapid proliferation of cancer cells, and can promote the process of glycometabolism reprogramming (91). Metabolic reprogramming is one of the important hallmarks of tumor cells, which are different from normal cells. Even in the presence of abundant oxygen, ~80% of tumor cells metabolize glucose and produce ATP mainly through aerobic glycolysis, also known as the Warburg effect. In addition to the rapid production of energy, glycolysis can also produce a large number of metabolic intermediates, which can be used to synthesize biological macromolecules needed for the rapid growth of tumors, including nucleotides, fatty acids, and proteins.

Glucose transporter (GLUT) is the main carrier of glucose uptake by cells. When glucose enters the cell, GLUT transports allosteric to transport glucose into the cell to support the high glycolysis rate. It has been reported that high glucose could promote the expression of vascular endothelial growth factor (VEGF)/VEGFR and the process of (epithelial-mesenchymal transition) EMT by regulating the expression of ERα/GLUT4, thereby promoting the proliferation and invasion of endometrial cancer cells (92). In addition, high glucose can increase the activity of glucose uptake and glycolysis by regulating AMPK/mTOR/S6 and MAPK pathways, thereby leading to increased invasiveness of endometrial cancer cells. Moreover, high glucose can promote the proliferation of endometrial cancer cells by activating STAT3 expression, which can be inhibited by metformin (93). The activity of glucose metabolism is closely related to the concentration of glucose outside the cell. However, it is not clear how cells perceive external glucose levels and regulate glycolysis pathway activity. AMPK, an adenylate-activated protein kinase, is a key protein for the perception of extracellular glucose concentration (94). It was reported that high extracellular glucose levels regulate the protein level of CARM1 by reducing AMPK phosphorylation, thereby inhibiting GAPDH methylation, which further promotes the activity of the GAPDH enzyme and glycolysis pathway (95). This study reveals the mechanism by which cells perceive extracellular glucose levels and regulate the rate of glycolysis, helping to elucidate the mechanism by which cancer cells perceive and utilize glucose.

Pyruvate kinase isozymes M2 (PKM2) is a key metabolic enzyme that promotes glycolysis and plays an important role in tumorigenesis through the Warburg effect. It has been reported that high glucose could promote the abnormal expression of PKM2. Overexpression of PKM2 could promote the accumulation of glycolysis intermediates (pyruvate and lactic acid), provide precursors for the synthesis of biomacromolecules, and lead to cell proliferation and tumorigenesis (96). Lactic acid is a key metabolite of glycolysis in cancer cells. The accumulation of extracellular lactic acid has an important impact on the metabolism of cancer cells and the transformation of non-cancer cells into cancer cells, including metabolic reprogramming, tumor inflammation, and angiogenesis. Recent studies have found that lactic acid produced by glycolysis of cancer cells can inhibit the function of macrophages through a hypoxia-inducible factor-mediated mechanism, induce the transformation of anti-tumor type M1 macrophages into M2, and promote the invasion and migration of cancer cells (97). Interestingly, M2 macrophages are the predominant tumor-associated macrophages in endometrial cancer and play an important role in the occurrence and development of endometrial cancer (98). Monocarboxylate Transporter 1 (MCT1) is an important protein for lactic acid and pyruvate uptake by cells, while MCT4 is an important protein for cell transport of lactic acid and pyruvate, which play an important role in regulating lactic acid metabolism. It has been reported that MCT1 was an independent prognostic biomarker in endometrial cancer (99). A recent study reported that the use of monocarboxylic acid transporter (MCT) inhibitors can reverse the inhibition of lactic acid on macrophage lysosomes (100). Therefore, MCT1 inhibition may have potential as a treatment for endometrial cancer. It is suggested that the enhancement of glycolysis activity and metabolites, such as lactic acid, in endometrial cancer under a high glucose environment may lead to acidification of the tumor microenvironment. The acidic environment may be perceived by tumor-related macrophages, which depend on the monocarboxylic acid transporter 1 (MCT1) pathway to induce macrophage transformation from M1 to M2, thus accelerating the progress of endometrial cancer. These studies suggested that managing hyperglycemia or targeting glycometabolism may be a potential therapeutic strategy for endometrial cancer.

### Effect of Insulin Resistance on Endometrial Cancer

Insulin resistance and hyperinsulinemia are important characteristics of obesity and diabetes. The expression of insulin and IGF-1 was significantly increased in diabetic patients, and high insulin levels were an independent factor of endometrial cancer (28, 101, 102). High insulin and IGF-1/2 levels in diabetic patients can accelerate the transformation of androstenedione into estrogen by aromatase and increase estrogen levels by inhibiting the synthesis of SHBG. Long-term estrogen stimulation without progesterone antagonism can cause endometrial dysplasia or even malignant transformation (103). It has been reported that after adjusting for BMI, age and histological type, the high expression of IR/IGF-1R is closely related to prognostic high-risk factors, such as the progression of endometrial cancer and lymph node infiltration (104).

Insulin can activate the phosphoinositide 3-kinase (PI3K)/AKT or mitogen-activated protein kinase (MAPK)/extracellular signaled regulated kinase ERK signaling pathway through binding to IR/IGF-1R to promote EMT of endometrial cancer, which leads to increased proliferation and invasion of endometrial cancer cells, inhibits apoptosis of cancer cells and promotes angiogenesis of tumors (105). In addition, insulin could upregulate the expression of vascular endothelial growth factor, thereby stimulating angiogenesis, which is closely related to the occurrence and development of tumors (106). Recent studies have reported that insulin signal-dependent phosphorylation initiates glucose metabolism prior to glucose transport; thus, the metabolism of glucose is diverted to a specific direction of glycolysis (107). In this way, insulin signaling plays a key role in glycometabolism reprogramming, and insulin resistance can promote the occurrence and development of endometrial cancer through an indirect pathway or ligand-receptor direct pathway.

IR-A has high affinity for insulin and IGF-II but binds IGF-I with low affinity. IGF-1R has high affinity for IGFs. However, blocking IGF-IR and IR does not completely prevent the growth stimulation of insulin-like growth factor or insulin on cancer cells, suggesting that other receptors may be involved in complex signal transduction systems. Our previous studies found and confirmed for the first time the expression of Hybrid-R in endometrial cancer, which can promote the proliferation and inhibit apoptosis of endometrial cancer cells through the MAPK/ERK signaling pathway (22). Hybrid-R is expected to provide a new therapeutic target and strategy for the precise treatment of endometrial cancer patients with insulin resistance and hyperinsulinemia.

### Metformin in the Treatment of Endometrial Cancer

Metformin, an insulin sensitizer, is considered a potential anticancer drug. Our previous clinical studies have found that metformin combined with progesterone treatment can significantly improve the efficacy of endometrial cancer patients with poor progesterone treatment (108). It has been reported that plasma hyperglycemia and high levels of IGF-1 in patients with endometrial cancer can be reversed by conventional doses of metformin (109). Metformin can significantly inhibit the proliferation of endometrial cancer cells, which may be related to the activation of AMPK signaling and the inhibition of the mTOR signaling pathway (110). In contrast, some studies have shown that metformin does not reduce the risk of endometrial cancer, nor can it improve the overall survival of patients (111). Correspondingly, studies have found that metformin does not affect the PI3K-Akt-mTOR and insulin signaling pathways and has no effect on weight loss (112). It is of great clinical significance to further study the mechanism of abnormal glucose metabolism promoting endometrial cancer, reveal the key molecule of endometrial cancer caused by metabolic diseases, and find new effective preventive or anti-cancer drugs that can replace metformin.

## Other Types OF Diseases OF Metabolic Syndrome And Endometrial Cancer

Dyslipidemia is closely related to the incidence of various cancers (113). It was reported that patients with hyperglycemia, hyperlipidemia and hypertension are twice as likely to develop endometrial cancer as normal people (114). The BMI of patients was positively correlated with serum palmitic acid, oleic acid and stearic acid levels. The increase of free fatty acids in obese patients can indirectly promote the proliferation of endometrial cancer cells by increasing the level of estradiol (115). The BMI of patients with endometrial cancer was significantly higher than that of normal controls. At present, there are many studies on the relationship between endometrial cancer and obesity and diabetes, but there are few studies on the relationship between endometrial cancer and hypertension and lipid disorders. The possible mechanisms of abnormal blood lipids associated with the risk of endometrial cancer are as follows: activation of fatty acid and amino hexose pathways leads to the production of reactive oxygen species (ROS) in mitochondria, which induces oxidative stress in cells. Excessive aggregation of ROS clusters interacts with lipids, proteins and DNA in cells, causing changes in membrane and enzyme functions, inducing cell damage, and ultimately leading to tumorigenesis (116). Recent studies have reported that serum cholesterol is elevated in obese people. Cholesterol activates the transcriptional activity of endometrial cancer cells through an ER-dependent pathway and promotes the proliferation of endometrial cancer cells (117). Moreover, recent studies have found that elevated circulating free fatty acids may be an important factor in linking obesity and tumorigenesis, which can promote the proliferation and invasion of breast cancer cells through ERα signaling and the mTOR signaling pathway (118). It has been reported that A-FABP released from adipose tissue can promote the dryness and invasiveness of breast cancer cells (119). Taken together, abnormal lipid metabolism, especially elevated free fatty acids, is closely related to the progression of endometrial cancer. Can fatty acids participate in the occurrence and development of endometrial cancer through an ER-dependent pathway remains to be thoroughly characterized.

Hypothyroidism is a type of reduced metabolic syndrome caused by a decrease in thyroid hormone synthesis and secretion or by inadequate physiological effects. Brinton et al. indicated that EC is related to previous diagnoses of thyroid diseases (RR = 1.52, 95% CI 1.17–1.98) (120). It has been reported that the incidence of hypothyroidism in EC patients is significantly increased. Serum TSH level before treatment is an independent risk factor for poor prognosis of EC (121). Additionally, elevated TSH levels have been reported to increase the incidence of MS (122). At present, there are few studies on thyroid function and endometrial cancer. Several studies have shown that hypothyroidism is closely related to MS, PCOS, elevated serum leptin levels, and dyslipidemia. Hypothyroidism may promote the occurrence and development of endometrial cancer by indirectly increasing the risk factors of endometrial cancer or through direct interaction (123). Studies examining the relationship between thyroid function (TSH level) and endometrial cancer will provide new insights into the mechanism of endometrial cancer.

## Conclusion

**Figure 2:**
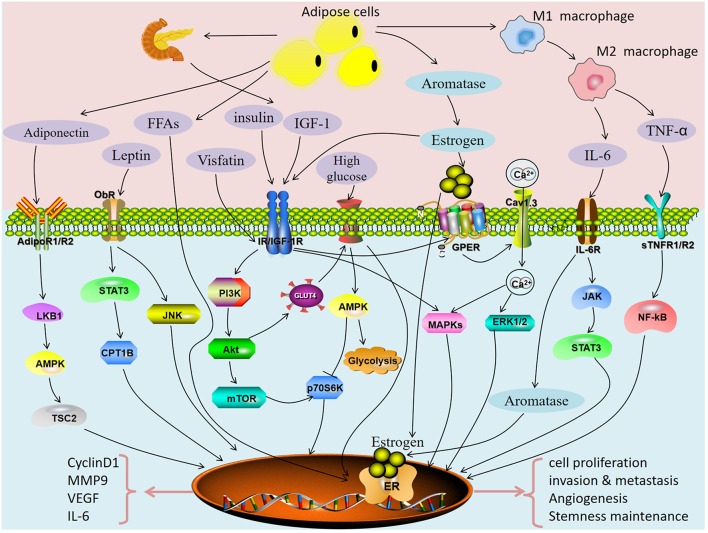
Potential pathways directly linking metabolic syndrome with endometrial cancer.

Metabolic syndrome is a complex disorder defined by a cluster of metabolic risk factors that includes insulin resistance, hyperinsulinemia, impaired glucose tolerance, type 2 diabetes mellitus, dyslipidemia, and visceral obesity. Obesity, diabetes and hypertension are the metabolic triad of endometrial cancer. There was a very high prevalence of metabolic syndrome in women newly diagnosed with endometrial cancer. In this article, we review potential pathways directly linking metabolic syndrome with cancer (Figure 2). Obesity-related insulin resistance, leptin and lactone levels are closely related to the occurrence and development of endometrial cancer. Decreased serum adiponectin levels and increased chronic inflammation in obese patients are important factors for increasing the risk of endometrial cancer. Obesity and diabetes have many common pathological characteristics: insulin resistance (hyperinsulinemia), abnormal fat metabolism (elevated leptin, decreased adiponectin), hyperglycemia, hyperlipidemia and chronic inflammation. Many studies have reported that these characteristics can promote the occurrence and development of endometrial cancer by directly acting on tumor cells or regulating the tumor microenvironment. Therefore, there is an urgent need to intervene in chronic diseases related to metabolic syndrome to reduce the incidence of endometrial cancer. A commentary by MacKintosh et al. indicated obese undergoing bariatric surgery or medical weight loss management could reduce the risk of endometrial cancer and hyperplastic abnormalities of the endometrium may be reversible through weight loss (124). Studies have assessed the effects of obesity and weight-loss surgery on endometrial morphology and molecular signaling pathways in endometrial cancer. It was found that insulin resistance (HbA1c, HOMA-IR) and inflammation (hsCRP, IL-6) circulating biomarkers decreased, while reproductive biomarkers (LH, FSH, SHBG) increased significantly (125). C-peptide, insulin, C-reactive protein, leptin, IL-1Ralpha, and IL-6 decreased significantly, while SHBG, IGFBP1, and adiponectin increased significantly in weight-loss interventions in endometrial cancer (126). Thus, bariatric surgery may reduce the risk of endometrial cancer by improving obesity-induced inflammation. However, the role of bariatric surgery in the treatment of endometrial hyperplasia still need conclusive or convincing evidence. Recently, seven markers based on the BMI-sensitive pathway of insulin resistance, adipoR1, adipoR2, ObR, IRβ, IRS-1, IGF-1R, and IGF-2R, have been proposed to develop a new molecular typing system for endometrial cancer. However, no effective molecular typing system and molecular typing markers have been found for endometrial cancer (127). A research proposed a pragmatic endometrial cancer risk prediction model, which included obesity, reproduction, insulin resistance, and genetic risk. This model plays an important role in identifying individuals at high risk of endometrial cancer and guiding preventive treatment of specific disease targets (128). Except for obesity, reproduction, insulin resistance, and genetic risk, the other metabolic syndrome related high risk factors for endometrial cancer as mentioned in the text should also be considered and a large prospective cohort of asymptomatic women is required. Similarly, studies incorporating biomarkers (adiponectin, estradiol, interleukin-1 receptor antagonist, tumor necrosis factor-and triglyceride) into the risk prediction model of endometrial cancer have found that they can modestly improve the predictive ability of endometrial cancer (129). Despite different studies, new molecular markers have been reported, but to date, no reliable molecular markers have been applied to clinical molecular typing. Our recent study found that serum total calcium may be a more sensitive metabolic syndrome parameter than hyperlipidemia in patients with endometrioid cancer (130).

Therefore, it is necessary to screen new markers based on systemic metabolomic changes and to reveal new molecular typing methods and risk prognostic models of endometrial cancer based on serum metabolomic changes. With the frequent application of proteomics, metabolomics, and transcriptomes, the role of key molecules in identifying metabolic syndrome-related diseases in endometrial cancer is of great significance for the early prevention and treatment of endometrial cancer. In conclusion, endometrial cancer is a type of metabolic disease-related tumor. Elucidating the specific roles and mechanisms of metabolic syndrome-related diseases in endometrial cancer is expected to provide a new target for the early prevention and treatment of endometrial cancer. Although the study of the association between metabolic syndrome and cancer may provide an effective therapeutic target for endometrial cancer, improving lifestyle is still the most important component in preventing the morbidity and mortality of endometrial cancer associated with metabolic syndrome. Further *in vivo* and clinical studies are needed to investigate the therapeutic targeting of the metabolic microenvironment in metabolic syndrome-related endometrial cancer.

## Author Contributions

XY and JW wrote and approved the final version of this manuscript.

### Conflict of Interest Statement

The authors declare that the research was conducted in the absence of any commercial or financial relationships that could be construed as a potential conflict of interest.
